# Ti insertion in the *M*Te_5_ (*M* = Zr, Hf) structure type: Hf_0.78_Ti_0.22_Te_5_

**DOI:** 10.1107/S1600536812006691

**Published:** 2012-02-17

**Authors:** Jaemin Yu, Hoseop Yun

**Affiliations:** a Division of Energy Systems Research and Department of Chemistry, Ajou University, Suwon 443-749, Republic of Korea

## Abstract

The group 5 mixed-metal telluride, Hf_0.78_Ti_0.22_Te_5_ (hafnium titanium penta­telluride), is isostructural with the binary phases HfTe_5_ and ZrTe_5_ and forms a layered structure extending parallel to (010). The layers are made up from chains of bicapped metal-centered trigonal prisms and zigzag Te chains. The metal site (site symmetry *m*2*m*) is occupied by statistically disordered Hf [78.1 (5)%] and Ti [21.9 (5)%]. In addition to the regular Te—Te pair [2.7448 (13) Å] forming the short base of the equilateral triangle of the trigonal prism, an inter­mediate Te⋯Te separation [2.9129 (9) Å] is also found. The classical charge balance of the compound can be described as [*M* ^4+^][Te^2−^][Te_2_^2−^][Te_2_^0^] (*M* = Hf, Ti). The individual metal content can vary in different crystals, apparently forming a random substitutional solid solution (Hf_1-*x*_Ti_*x*_)Te_5_, with 0.15 ≤ *x* ≤ 0.22.

## Related literature

For the synthesis and structure of HfTe_5_ and ZrTe_5_, see: Brattås & Kjekshus (1971 ▶); Furuseth *et al.* (1973 ▶, 1975 ▶). For properties of HfTe_5_ and ZrTe_5_, see: DiSalvo *et al.* (1981 ▶). For extensive Te⋯Te inter­actions in metal tellurides, see: Pell & Ibers (1996 ▶); Mar & Ibers (1993 ▶).

## Experimental

### 

#### Crystal data

Hf_0.78_Ti_0.22_Te_5_
*M* *_r_* = 787.76
Orthorhombic, 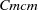

*a* = 3.9595 (3) Å
*b* = 14.4350 (13) Å
*c* = 13.7062 (9) Å
*V* = 783.39 (10) Å^3^
*Z* = 4
Mo *K*α radiation
μ = 28.76 mm^−1^
*T* = 290 K
0.30 × 0.04 × 0.02 mm

#### Data collection

Rigaku R-AXIS RAPID diffractometer
Absorption correction: multi-scan (*NUMABS*; Higashi, 2000 ▶) *T*_min_ = 0.263, *T*_max_ = 1.000
3405 measured reflections
533 independent reflections
516 reflections with *I* > 2σ(*I*)
*R*_int_ = 0.059

#### Refinement

*R*[*F* ^2^ > 2σ(*F* ^2^)] = 0.029
*wR*(*F* ^2^) = 0.068
*S* = 1.16
533 reflections
23 parameters
Δρ_max_ = 2.38 e Å^−3^
Δρ_min_ = −2.07 e Å^−3^

### 

Data collection: *RAPID-AUTO* (Rigaku, 2006 ▶); cell refinement: *RAPID-AUTO*; data reduction: *RAPID-AUTO*; program(s) used to solve structure: *SHELXS97* (Sheldrick, 2008 ▶); program(s) used to refine structure: *SHELXL97* (Sheldrick, 2008 ▶); molecular graphics: *DIAMOND3* (Brandenburg, 1999 ▶); software used to prepare material for publication: *WinGX* (Farrugia, 1999 ▶).

## Supplementary Material

Crystal structure: contains datablock(s) global, I. DOI: 10.1107/S1600536812006691/wm2588sup1.cif

Structure factors: contains datablock(s) I. DOI: 10.1107/S1600536812006691/wm2588Isup2.hkl

Additional supplementary materials:  crystallographic information; 3D view; checkCIF report

## Figures and Tables

**Table:** *M* = Hf or Ti

*M*—Te2^i^	2.9251 (6)
*M*—Te1^ii^	2.9446 (8)
*M*—Te3	2.9498 (7)

**Table:** 

Te3^ii^—Te3—Te3^iii^	85.63 (3)

Symmetry codes: (i) 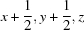; (ii) 
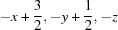
; (iii) 
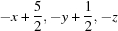
.

**Fig. 1.**
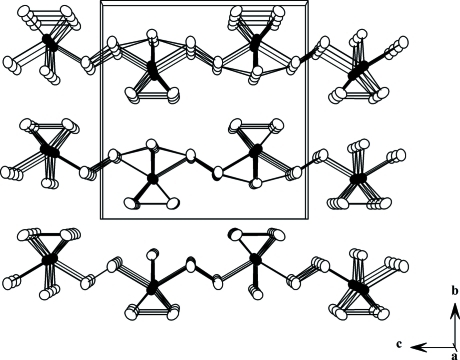
A perspective view of Hf0.78Ti0.22Te5 down the a axis showing the stacking of the layers. Filled and open circles represent metals and S atoms, respectively. The displacement ellipsoids are drawn at the 90% probability level.

**Fig. 2.**
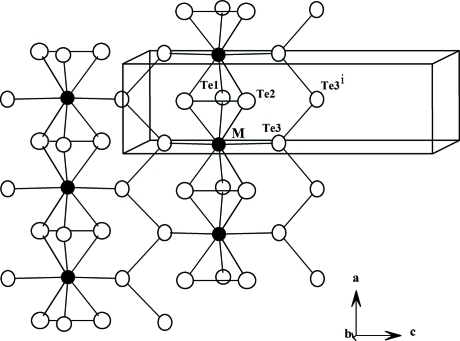
A view of the layer of Hf0.78Ti0.22Te5. Anisotropic displacement ellipsoids are drawn at the 90% probability level. Atoms are as marked as in Fig. 1. [Symmetry code: (i) 1/2 + x, 1/2 - y, 1 - z].

## supplementary crystallographic information

### Comment

The existence of the binary pentatellurides, *M*Te_5_ (*M* = Zr, Hf) was established in 1971 by Brattås & Kjekshus. These compounds have been the most chalcogen-rich stoichiometric transition metal binary chalcogenides since then. These phases are important not only because of their unusually high chalcogen contents but because of the anomalies in their transport properties (DiSalvo *et al.*, 1981). The Ti analogue of this phase is desired to study the relationship between structure and properties. However, efforts to find TiTe_5_ have not been successful despite numerous attempts (Furuseth *et al.*, 1973). Indeed, no Ti analogue of the *M*Te_8_ polyhedra found in *M*Te_5_ (Furuseth *et al.*, 1975) has been observed up to now. During attempts to synthesize new metal tellurides, we found the new Ti-containing mixed-metallic phase Hf_0.78_Ti_0.22_Te_5_.

The title compound is isostructural with HfTe_5_ and ZrTe_5_. Detailed descriptions of this structural type have been reported previously (Furuseth *et al.*, 1973). A view of the structure down the *a* axis is given in Fig. 1, which shows the layered nature parallel to (010). The structure is composed of chains made up from bicapped trigonal prismatic *M*Te_8_ units. The metal (*M*) site occupied by statistically disordered Hf (78.1 (5) %) and Ti (21.9 (5) %) is surrounded by eight Te atoms. Three crystallographically independent Te sites are found in the title compound. Both Te1 (site symmetry *m*2*m*) and Te2 (site symmetry *m*..) are at the corners of a triangular prism and they are bridging the *M* atoms to form a chain. Two Te3 (site symmetry *m*..) atoms outside the rectangular faces of the prism are connected to the neighboring Te3 to form the infinite zigzag Te chain. Finally, the *M*Te_5_ layer is formed by the alternate linking of these chains (Fig. 2).

The structure shows a wide range of Te···Te interactions. In the prism, the Te2—Te2 pair (2.7448 (13) Å) forming the short base of the equilateral triangle exhibits a regular Te—Te bond, Te_2_^2-^ (*e.g.* discussed by Pell & Ibers, 1996). In addition, an intermediate Te3···Te3 separation (2.9129 (9) Å) is indicative of a weak single bond (Mar & Ibers, 1993) but we assign an oxidation state of 0 for Te3 (^1^_∞_[Te^0^]). The classical charge balance of the compound can be described as [*M*^4+^][Te^2-^][Te_2_^2-^][Te_2_^0^] (*M*=Hf, Ti).

Structury analysis of three different crystals from the same reaction tube showed that the metal content can vary, apparently forming a random substitutional solid solution (Hf_1-_*_x_*Ti*_x_*)Te_5_, 0.15 ≤*x*≤ 0.22.

### Experimental

The title compound, Hf_0.78_Ti_0.22_Te_5_, was prepared by the reaction of the elements with the use of the reactive halide-flux technique. Hf powder (CERAC 99.8%), Ti powder (CERAC 99.5%), and Te powder (CERAC 99.95%) were mixed in a fused silica tube in a molar ratio of Hf: Ti: Te = 1: 1: 10 and then CsCl (CERAC 99.9%) was added in a weight ratio of HfTiTe_10_: CsCl = 1: 2. The tube was evacuated to 0.133 Pa, sealed and heated gradually (50 K/h) to 650 K, where it was kept for 72 h. The tube was then cooled to room temperature at the rate of 3 K/h. The excess halide was removed with distilled water and dark block-shaped crystals were obtained. The crystals are stable in air and water. A qualitative X-ray fluorescence analysis of the crystals indicated the presence of Hf, Ti, and Te. A quantitative XRF analysis indicated that the Hf: Ti ratio is 80: 20. The composition of the compound was determined by single-crystal X-ray diffraction.

### Refinement

The statistically disordered nature of the *M* site in the title compound was checked by refining the anisotropic displacement parameters (ADPs). When the model was refined assuming HfTe_5_ or TiTe_5_, the displacement parameters of the metal site were very large and small, respectively. In both cases the reliability indices were rather high (wR2 > 0.127). In the refined mixed-metal model, the ADPs of the metal atoms are comparable with those of the other atoms and the residuals were reduced significantly (wR2 = 0.068). The remaining highest and lowest electron densities are found 0.81 and 0.77 Å from atom Te1.

### Crystal data

**Table:**

Hf_0.78_Ti_0.22_Te_5_	*F*(000) = 1284
*M**_r_* = 787.76	*D*_x_ = 6.679 Mg m^−^^3^
Orthorhombic, *C**m**c**m*	Mo *K*α radiation, λ = 0.71073 Å
Hall symbol: -C 2c 2	Cell parameters from 3264 reflections
*a* = 3.9595 (3) Å	θ = 3.2–27.5°
*b* = 14.4350 (13) Å	µ = 28.76 mm^−^^1^
*c* = 13.7062 (9) Å	*T* = 290 K
*V* = 783.39 (10) Å^3^	Needle, black
*Z* = 4	0.30 × 0.04 × 0.02 mm

### Data collection

**Table:**

Rigaku R-AXIS RAPID diffractometer	533 independent reflections
Radiation source: fine-focus sealed tube	516 reflections with *I* > 2σ(*I*)
Graphite monochromator	*R*_int_ = 0.059
ω scans	θ_max_ = 27.5°, θ_min_ = 2.8°
Absorption correction: multi-scan (*NUMABS*; Higashi, 2000)	*h* = −4→5
*T*_min_ = 0.263, *T*_max_ = 1.000	*k* = −18→18
3405 measured reflections	*l* = −17→17

### Refinement

**Table:**

Refinement on *F*^2^	Primary atom site location: structure-invariant direct methods
Least-squares matrix: full	Secondary atom site location: difference Fourier map
*R*[*F*^2^ > 2σ(*F*^2^)] = 0.029	*w* = 1/[σ^2^(*F*_o_^2^) + (0.0304*P*)^2^ + 7.5394*P*] where *P* = (*F*_o_^2^ + 2*F*_c_^2^)/3
*wR*(*F*^2^) = 0.068	(Δ/σ)_max_ < 0.001
*S* = 1.16	Δρ_max_ = 2.38 e Å^−^^3^
533 reflections	Δρ_min_ = −2.07 e Å^−^^3^
23 parameters	Extinction correction: *SHELXL97* (Sheldrick, 2008)
0 restraints	Extinction coefficient: 0.00109 (14)

### Special details

**Table:**

Geometry. All s.u.'s (except the s.u. in the dihedral angle between two l.s. planes) are estimated using the full covariance matrix. The cell s.u.'s are taken into account individually in the estimation of s.u.'s in distances, angles and torsion angles; correlations between s.u.'s in cell parameters are only used when they are defined by crystal symmetry. An approximate (isotropic) treatment of cell s.u.'s is used for estimating s.u.'s involving l.s. planes.
Refinement. Refinement of *F*^2^ against ALL reflections. The weighted *R*-factor *wR* and goodness of fit *S* are based on *F*^2^, conventional *R*-factors *R* are based on *F*, with *F* set to zero for negative *F*^2^. The threshold expression of *F*^2^ > 2σ(*F*^2^) is used only for calculating *R*-factors(gt) *etc*. and is not relevant to the choice of reflections for refinement. *R*-factors based on *F*^2^ are statistically about twice as large as those based on *F*, and *R*- factors based on ALL data will be even larger.

### Fractional atomic coordinates and isotropic or equivalent isotropic displacement parameters (Å^2^)

**Table:**

	*x*	*y*	*z*	*U*_iso_*/*U*_eq_	Occ. (<1)
Hf	1	0.31435 (5)	0.25	0.0109 (3)	0.781 (5)
Ti	1	0.31435 (5)	0.25	0.0109 (3)	0.219 (5)
Te1	1	0.33665 (6)	−0.25	0.0128 (3)	
Te2	1	−0.07070 (5)	0.14987 (5)	0.0169 (3)	
Te3	1	0.20952 (5)	0.06526 (4)	0.0154 (2)	

### Atomic displacement parameters (Å^2^)

**Table:**

	*U*^11^	*U*^22^	*U*^33^	*U*^12^	*U*^13^	*U*^23^
Hf	0.0103 (4)	0.0122 (4)	0.0102 (4)	0	0	0
Ti	0.0103 (4)	0.0122 (4)	0.0102 (4)	0	0	0
Te1	0.0129 (4)	0.0121 (5)	0.0134 (4)	0	0	0
Te2	0.0164 (4)	0.0164 (4)	0.0178 (4)	0	0	0.0051 (2)
Te3	0.0155 (4)	0.0190 (4)	0.0116 (3)	0	0	−0.0010 (2)

### Geometric parameters (Å, º)

**Table:**

M—Te2^i^	2.9251 (6)	Te1—M^v^	2.9446 (8)
M—Te2^ii^	2.9251 (6)	Te1—M^vi^	2.9446 (8)
M—Te2^iii^	2.9251 (6)	Te1—M^vi^	2.9446 (8)
M—Te2^iv^	2.9251 (6)	Te2—Te2^vii^	2.7448 (13)
M—Te1^v^	2.9446 (8)	Te2—M^viii^	2.9251 (6)
M—Te1^vi^	2.9446 (8)	Te2—M^viii^	2.9251 (6)
M—Te3	2.9498 (7)	Te2—M^ix^	2.9251 (6)
M—Te3^vii^	2.9498 (7)	Te2—M^ix^	2.9251 (6)
Te1—M^v^	2.9446 (8)	Te3—Te3^v^	2.9129 (9)
			
Te2^i^—M—Te2^ii^	55.96 (3)	Te2^iv^—M—Te3^vii^	83.583 (16)
Te2^i^—M—Te2^iii^	110.88 (3)	Te1^v^—M—Te3^vii^	67.683 (15)
Te2^ii^—M—Te2^iii^	85.19 (2)	Te1^vi^—M—Te3^vii^	67.683 (15)
Te2^i^—M—Te2^iv^	85.19 (2)	Te3—M—Te3^vii^	118.27 (3)
Te2^ii^—M—Te2^iv^	110.88 (3)	M^v^—Te1—M^v^	0.000 (18)
Te2^iii^—M—Te2^iv^	55.96 (3)	M^v^—Te1—M^vi^	84.50 (3)
Te2^i^—M—Te1^v^	151.040 (16)	M^v^—Te1—M^vi^	84.50 (3)
Te2^ii^—M—Te1^v^	151.040 (16)	M^v^—Te1—M^vi^	84.50 (3)
Te2^iii^—M—Te1^v^	87.986 (16)	M^v^—Te1—M^vi^	84.50 (3)
Te2^iv^—M—Te1^v^	87.986 (16)	M^vi^—Te1—M^vi^	0
Te2^i^—M—Te1^vi^	87.986 (16)	Te2^vii^—Te2—M^viii^	62.019 (13)
Te2^ii^—M—Te1^vi^	87.986 (16)	Te2^vii^—Te2—M^viii^	62.019 (13)
Te2^iii^—M—Te1^vi^	151.040 (16)	M^viii^—Te2—M^viii^	0.00 (2)
Te2^iv^—M—Te1^vi^	151.040 (16)	Te2^vii^—Te2—M^ix^	62.019 (13)
Te1^v^—M—Te1^vi^	84.49 (3)	M^viii^—Te2—M^ix^	85.19 (2)
Te2^i^—M—Te3	133.927 (10)	M^viii^—Te2—M^ix^	85.19 (2)
Te2^ii^—M—Te3	83.584 (16)	Te2^vii^—Te2—M^ix^	62.019 (13)
Te2^iii^—M—Te3	83.583 (16)	M^viii^—Te2—M^ix^	85.19 (2)
Te2^iv^—M—Te3	133.927 (10)	M^viii^—Te2—M^ix^	85.19 (2)
Te1^v^—M—Te3	67.683 (15)	M^ix^—Te2—M^ix^	0.00 (2)
Te1^vi^—M—Te3	67.683 (15)	Te3^v^—Te3—Te3^vi^	85.63 (3)
Te2^i^—M—Te3^vii^	83.583 (16)	Te3^v^—Te3—M	108.74 (3)
Te2^ii^—M—Te3^vii^	133.927 (10)	Te3^vi^—Te3—M	108.74 (3)
Te2^iii^—M—Te3^vii^	133.927 (10)		

Symmetry codes: (i) *x*+1/2, *y*+1/2, −*z*+1/2; (ii) *x*+1/2, *y*+1/2, *z*; (iii) *x*−1/2, *y*+1/2, *z*; (iv) *x*−1/2, *y*+1/2, −*z*+1/2; (v) −*x*+3/2, −*y*+1/2, −*z*; (vi) −*x*+5/2, −*y*+1/2, −*z*; (vii) *x*, *y*, −*z*+1/2; (viii) *x*−1/2, *y*−1/2, *z*; (ix) *x*+1/2, *y*−1/2, *z*.
